# Protective Effects of *Fusarium venenatum*-Based Mycoprotein against Metabolic Dysfunction-Associated Steatohepatitis via the Gut-Liver Axis

**DOI:** 10.4014/jmb.2602.02028

**Published:** 2026-04-21

**Authors:** Daniel Junpyo Lee, Daye Mun, Min-Geun Kang, Anna Kang, Youbin Choi, Eunsol Seo, Seon-hui Son, Jihyun Yoon, Arthur Junghun Kim, Min-Jin Kwak, Woo Kyun Kim, Minho Song, Sangnam Oh, Younghoon Kim

**Affiliations:** 1 Department of Agricultural Biotechnology and Research Institute of Agriculture and Life Science, Seoul National University, Seoul 08826, Republic of Korea; 2 Division of Animal Bioscience and Integrated Biotechnology, College of Agriculture and Life Sciences, Gyeongsang National University, Jinju 52828, Republic of Korea; 3 Department of Forest Products and Biotechnology, Kookmin University, Seoul 02707, Korea; 4 Department of Poultry Science, University of Georgia, Athens, GA 30602, USA; 5 Department of Animal Science and Biotechnology, Chungnam National University, Daejeon 34134, Republic of Korea; 6 Department of Food and Nutrition, Jeonju University, Jeonju 55069, Republic of Korea

**Keywords:** Metabolic dysfunction-associated steatohepatitis (MASH), *Fusarium venenatum*, Mycoprotein, Gut-liver axis, Intestinal barrier integrity

## Abstract

Metabolic dysfunction-associated steatohepatitis (MASH) is a progressive subtype of metabolic dysfunction-associated steatotic liver disease, and gut microbiota dysbiosis has been implicated in promoting bacterial translocation and lipopolysaccharide influx into the portal circulation, driving hepatic inflammation. In this study, we evaluated the protective effects of *Fusarium venenatum*-based mycoprotein against MASH development in two diet-induced murine models. To induce MASH, mice were fed either a high-fat, high-fructose, choline-deficient diet or, for a more severe phenotype, a diet deficient in both choline and methionine. Mice were fed the respective diet for three weeks while receiving oral *F. venenatum* supplementation. In both models, supplementation significantly attenuated hepatic steatosis, fibrosis, and inflammation, accompanied by reduced hepatic expression of pro-inflammatory cytokines. In the intestine, tight junction-related genes were upregulated, while pro-inflammatory cytokine gene expression was suppressed. Fecal metabolomics revealed increased cholesterol excretion, indicating reduced cholesterol absorption. Gut microbiota analysis demonstrated a reduction in Proteobacteria and Deferribacterota and an enrichment of Verrucomicrobiota, particularly *Akkermansia*, in *F. venenatum*-treated mice. These findings suggest that *F. venenatum*-based mycoprotein may attenuate MASH progression by improving intestinal barrier function, increasing cholesterol excretion, and reshaping the gut microbial community, collectively contributing to favorable modulation of the gut-liver axis.

## Introduction

Metabolic dysfunction-associated steatotic liver disease (MASLD), formerly known as non-alcoholic fatty liver disease (NAFLD), has emerged as the most prevalent chronic liver disorder worldwide, affecting approximately 32% of the global population [1-3]. MASLD is tightly linked to obesity and metabolic syndromes, largely driven by Western-style diets [4]. MASLD encompasses a histological spectrum of liver disorders, ranging from simple steatosis to steatohepatitis, fibrosis, cirrhosis, and ultimately hepatocellular carcinoma, occurring in the absence of significant alcohol consumption [5, 6]. Among these stages, metabolic dysfunction-associated steatohepatitis (MASH), previously referred to as non-alcoholic steatohepatitis (NASH), is a progressive form of MASLD. MASH is histologically characterized by hepatic steatosis accompanied by hepatocellular ballooning, lobular inflammation, and varying degrees of fibrosis [2, 7]. While simple steatosis confers a low risk of progression, MASH substantially increases the risk of cirrhosis, hepatocellular carcinoma, and liver-related mortality [8].

The gastrointestinal tract hosts a complex and dynamic microbial ecosystem that plays a pivotal role in maintaining host physiology and metabolic homeostasis [9-14]. Gut dysbiosis is increasingly implicated in the initiation and progression of liver diseases, including MASH [7, 15]. The gut and liver are anatomically and functionally connected via the portal circulation, facilitating the direct delivery of gut-derived metabolites and microbial components to the liver [16]. Among these, lipopolysaccharides (LPS), a major component of the outer membrane of Gram-negative bacteria, is widely recognized as a potent stimulator of inflammatory responses [17-20]. Under conditions of dysbiosis and increased intestinal permeability, endotoxins such as LPS translocate into the portal bloodstream and reach the liver, where they trigger hepatic inflammation [21]. Within the hepatic environment, LPS and other pathogen-associated molecular patterns (PAMPs) activate Toll-like receptor 4 (TLR4) signaling in Kupffer cells and hepatic stellate cells, triggering NF-κB signaling pathways that lead to the production of pro-inflammatory cytokines, and contributing to hepatocyte injury, and activation of fibrogenic responses [22, 23]. Chronic exposure to these inflammatory stimuli not only perpetuates hepatic damage but also promotes lipid accumulation in hepatocytes [24]. These mechanisms highlight the importance of intestinal barrier dysfunction and microbial translocation in the pathophysiology of MASH [25, 26].

*Fusarium venenatum*, a filamentous fungus, is widely recognized for its application in the production of mycoprotein. Mycoprotein derived from *F. venenatum* is characterized by its high protein and fiber content, low fat, and relatively low energy density [27]. Previous *in vitro* studies have suggested that the fibrous structure of *F. venenatum* can entrap digestive components such as amylase, lipase, and bile salts, thereby reducing the efficiency of starch hydrolysis and lipid digestion [28]. Furthermore, our recent findings demonstrated that dietary supplementation with *F. venenatum* attenuated hepatic lipid accumulation, improved intestinal barrier integrity, reduced systemic inflammation, and facilitated fecal cholesterol excretion [29]. These multifunctional effects position *F. venenatum* as a promising candidate for dietary intervention in MASH.

In this study, we aimed to evaluate the protective effects of *F. venenatum*-based mycoprotein against the development and progression of MASH. We conducted two independent experiments mouse experiments in which MASH was induced by dietary challenge. In the first experiment, a high-fat, high-fructose, choline-deficient (HCF) diet was used to induce MASH. In the second experiment, a more severe phenotype was elicited using a diet deficient in both choline and methionine (HMF diet). Through biological assays and multi-omics analysis, we investigated the potential of *F. venenatum*-based mycoprotein as a functional dietary strategy for mitigating MASH progression.

## Materials and Methods

### Preparation of *F. venenatum* Based Mycoprotein

The mycoprotein derived from *F. venenatum* used in this study was produced using the *F. venenatum* strain KACC No. 49797 (A3/5), which is the same strain employed in the commercial production of Quorn^TM^ for human consumption. This product has been granted Generally Recognized as Safe status by the U.S. Food and Drug Administration [30]. Fermentation was carried out in malt extract broth (BD Biosciences, USA), following Quorn^TM^ production procedures previously described by Finnigan and Wiebe [31, 32].

### Animals and Experimental Design

**Fig. 1:**
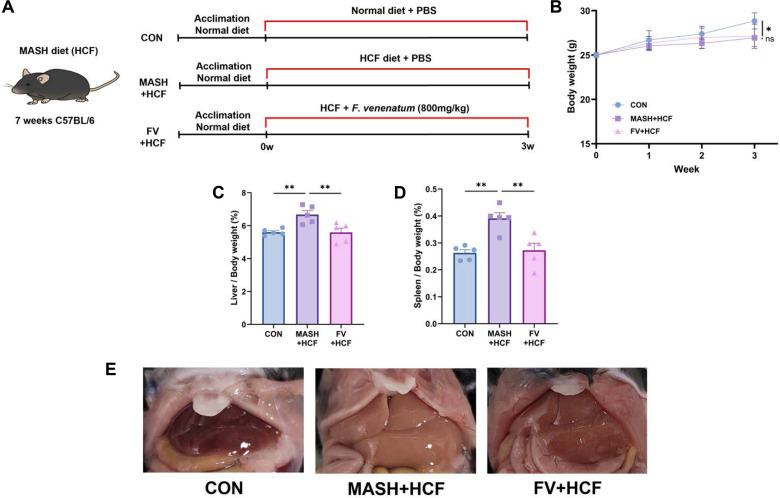
*F. venenatum* attenuated MASH progression in HCF diet-fed mice. (**A**) Experimental design. (**B**) Body weight change. (**C**) Liver weight. (**D**) Spleen weight. (**E**) Representative liver image. Data are expressed as means ± SEM. Statistical analysis was performed using one-way ANOVA. Differences were considered significant when *p*-value was below 0.05 (*), 0.01 (**), 0.001 (***), 0.0001 (****). CON group, received an oral administration of 200 μL of PBS along with a normal diet; MASH-HCF group, received an oral administration of 200 μl of PBS along with a HCF diet for MASH development; FV-HCF group, received an oral administration of *F. venenatum* at a dose of 800 mg/kg, suspended in 200 μl of PBS, along with a HCF diet for MASH development.

All animal experiments were conducted following review and approval by the Institutional Animal Care and Use Committee of Seoul National University SNU-220111-4. Seven-week-old male C57BL/6 mice were used in experiments. All mice were housed under controlled environmental conditions (12-h light/dark cycle, 55 ± 5% humidity, 22 ± 1°C) in individually ventilated cages, with ad libitum access to food and water throughout the experimental period. In the first study, MASH was induced by feeding the HCF diet. In the second study, the HMF diet was administered to induce a more severe form of MASH. The compositions of the diets used in these studies are shown in Table 1. The diets used in the present experiments were the same as those used in prior MASH induction studies [7]. In both studies, after a one-week acclimation period, mice were randomly assigned to three groups (n = 5 per group) based on body weight. The CON group received oral administration of 200 μL of PBS along with a normal diet; the MASH group received 200 μL of PBS along with a MASH diet; and the FV group received *F. venenatum*-based mycoprotein at a dose of 800 mg/kg, suspended in 200 μL of PBS, along with a MASH diet. The dosage of 800 mg/kg was adopted based on previous research [29]. The experiment lasted for 3 weeks. The experimental designs for both the HCF and HMF diet models are shown in Fig. 1A and Supplementary Fig. S1A.

### Sample Collection

The liver and spleen were weighed, and the liver-to-body weight and spleen-to-body weight ratios were calculated. Liver tissues were either fixed in 4% paraformaldehyde solution and stored at 4°C for histological analysis, or snap-frozen in liquid nitrogen and stored at -80°C for RNA extraction. Serum and colon samples were collected and stored at -80°C for further analysis. For fecal sample collection, sterile microtubes and autoclaved forceps were used to minimize exogenous microbial contamination. Fresh fecal pellets were obtained immediately after natural defecation, snap-frozen in liquid nitrogen, and stored at -80°C until DNA extraction.

### Serum Analysis

Serum samples were collected at week 3, and the levels of total cholesterol, triglycerides, aspartate transaminase (AST), and alanine transaminase (ALT) were measured. All biochemical analyses were performed using a Fuji DRI-CHEM Clinical Chemistry Analyzer FDC 3500 (Fujifilm, Japan).

### Extraction of Total Lipids from Hepatic Tissue

Total hepatic lipid content was measured according to a previously described method [7]. A total of 100 mg of homogenized liver tissue was combined with 2 mL of a hexane: isopropanol mixture (3:2, v/v) and incubated at 20°C with continuous rotation overnight. Following incubation, 1 mL of sodium sulfate solution was added, and the sample was vortexed and centrifuged at 400 × g for 10 min. The supernatant was transferred to a fresh vial. To maximize lipid extraction, an additional 1 mL of hexane was added to the residual pellet, followed by another overnight rotation, vortexing, and centrifugation under identical conditions. The supernatant from this second extraction was combined with the first. The pooled supernatant was evaporated, and the total lipid content was determined by weighing the dried residue. The dried lipid was subsequently dissolved in 1 mL of 1% Triton X-100 for further analysis. Hepatic total cholesterol and triglyceride levels were quantified using commercial assay kits (Embiel, Republic of Korea) according to the manufacturer’s instructions.

### Histological Analysis

Fixed liver tissues were stained with hematoxylin and eosin (H&E) and Sirius Red for histological evaluation. The nonalcoholic fatty liver disease activity score (NAS) was calculated using three histological components, including steatosis, hepatocyte ballooning, and lobular inflammation. Steatosis and lobular inflammation were each scored from 0 to 3, and hepatocyte ballooning from 0 to 2, yielding a total score ranging from 0 to 8. A NAS ≥ 5 was considered indicative of definite MASH, while a score ≤ 3 was classified as not MASH [33, 34].

### RNA Isolation and Quantitative Real-Time PCR (qRT-PCR) Analysis

Total RNA was extracted from liver and colon tissues using the RNeasy Plus Kit (Qiagen, Germany). cDNA synthesis was performed using the iScript cDNA Synthesis Kit (Bio-Rad, USA). qRT-PCR was conducted using the CFX96^TM^ Real-Time PCR System (Bio-Rad) and the SsoAdvanced^TM^ Universal SYBR*®* Green Supermix (Bio-Rad). In liver tissue, *Krt18* (encodes keratin 18), *Tgfb1* (transforming growth factor-β1), *Acta2* (alpha-smooth muscle actin), *Hmgcr* (3-hydroxy-3-methylglutaryl-CoA reductase), *Tnf* (tumor necrosis factor-α), and *Il1b* (interleukin-1β) were measured. In colon tissue, *Ocln* (occludin), *Cldn1* (claudin-1), *Tjp1* (zonula occludens-1), *Tnf* (tumor necrosis factor-α), and *Il1b* (interleukin-1β) were measured. All genes were normalized to *Hprt1* (hypoxanthine-guanine phosphoribosyltransferase 1). Each of the samples was technically replicated twice. Primers used in this study are listed in Table 2.

### Metabolomic Analysis

Metabolomic analysis was performed according to Lee's methods with slight modification [29]. Briefly, 40 mg of freeze-dried fecal sample was mixed with 1 mL of ice-cold methanol. Silica beads were added, and the mixture was homogenized using a bead beater with five cycles of 1 min of beating followed by 1 min on ice. The homogenized samples were then kept at -4°C for 30 min. After incubation, the samples were centrifuged at 15,000 × g for 5 min at 4°C, and the resulting supernatant was filtered through 0.2 μm pore size polyvinylidene fluoride (PVDF) syringe filters (Whatman, England). A 200 μL aliquot of the filtered supernatant was dried using a vacuum concentrator. For derivatization, the dried extract was treated with 30 μL of 20 mg/mL methoxyamine hydrochloride in pyridine (Sigma, USA) at 30°C for 90 min. Subsequently, 50 μL of N,O-Bis(trimethylsilyl)trifluoroacetamide (BSTFA; Sigma) was added and incubated at 60°C for 30 min. 10 μL of 1 mM fluoranthene (Sigma) was added to the extract as an internal standard.

GC-MS analysis was conducted using a Thermo Trace 1310 gas chromatograph (Thermo Fisher Scientific, USA) coupled to an ISQ LT single quadrupole mass spectrometer (Thermo Fisher Scientific). Metabolite separation was achieved using a DB-5MS capillary column with 60 m length, 0.2 mm i.d., and 0.25 μm film thickness (Agilent, USA). For sample injection, the inlet temperature was set to 300°C, and the injection was performed with a split ratio of 1:60 using a helium split flow of 7.5 mL/min. Metabolite separation was carried out under a constant helium flow rate of 1.5 mL/min using the following oven temperature program: an initial hold at 50°C for 2 min, followed by a ramp to 180°C at 5°C/min with an 8-min hold, then to 210°C at 2.5°C/min, and finally to 325°C at 5°C/min with a 10-min final hold. Mass spectral data were collected in full scan mode over an m/z range of 35 to 650 at a scan rate of five spectra/s. Electron impact ionization was applied, and the ion source temperature was set to 270°C. Spectral data were processed using Thermo Xcalibur software with automated peak detection. Metabolites were identified by matching mass spectra with the NIST Mass Spectral Search Program (version 2.0, USA). Further analyses were conducted using MetaboAnalyst 6.0 software.

### 16S rRNA Amplicon Sequencing and Microbiota Analysis

Fecal DNA was extracted using the PowerFecal DNA Isolation Kit (Qiagen, Germany). The V3-V4 region of the bacterial 16S rRNA gene was amplified via PCR and sequenced on the Illumina NextSeq platform (2 × 300 bp) (Illumina Inc., USA) by following the manufacturer's protocols. Raw sequencing reads were demultiplexed and processed through a series of quality control steps, including adapter removal, quality trimming, error correction, and chimera elimination. Amplicon sequence variants were generated using the Divisive Amplicon Denoising Algorithm 2 algorithm implemented within the QIIME 2 framework. Taxonomic assignment was performed using the SILVA database (version 138.1). Alpha and beta diversity metrics were calculated using QIIME 2 and visualized with GraphPad Prism (version 10.4.2). Compositional differences at the genus level were analyzed using the Statistical Analysis of Metagenomic Profiles (STAMP) software, applying Welch’s t-test (*p* < 0.05). The sequencing data generated in this study have been deposited in the NCBI Sequence Read Archive under accession number PRJNA1304152.

### Statistical Analysis

Statistical analyses were conducted using GraphPad Prism (version 10.4.2). Prior to group comparisons, data distribution was evaluated using the Shapiro–Wilk normality test. As all datasets satisfied the assumption of normality (*p* > 0.05), one-way analysis of variance (ANOVA) was performed, followed by Tukey’s post hoc test. Statistical significance was considered when the *p*-values were below 0.05 (*), 0.01 (**), 0.001 (***), and 0.0001 (****).

## Results

### Attenuation of MASH Pathology by *F. venenatum*-Based Mycoprotein in HCF and HMF Diet-Fed Mice

In the HCF model, mice were fed a high-fat, high-fructose, choline-deficient diet, to induce MASH. After 3 weeks of intervention, no significant difference in body weight was observed between the MASH-HCF and FV-HCF groups (Fig. 1B) (*p* = 0.9660). However, liver weight was significantly reduced in the FV-HCF group compared to the MASH-HCF group (Fig. 1C) (*p* = 0.0091). In addition, spleen weight, an indicator of systemic immune activation, was also significantly lower in the FV-HCF group compared to the MASH-HCF group (Fig. 1D) (*p* = 0.0038). Representative liver images revealed a pale and enlarged liver in the MASH-HCF group, whereas the FV-HCF group exhibited improved hepatic morphology (Fig. 1E).

Next, in the second experiment, a more severe MASH phenotype was induced using a high-fat, high-fructose, choline- and methionine-deficient (HMF) diet. Similarly, by week 3, both MASH-HMF and FV-HMF groups showed significantly lower body weights compared to the CON group (Supplementary Fig. S1B) (*p* < 0.0001 for MASH-HMF and *p* < 0.0001 for FV-HMF). Notably, the FV-HMF group maintained a significantly higher body weight than the MASH-HMF group (Supplementary Fig. S1B) (*p* = 0.0016). Liver weight was also significantly decreased in the FV-HMF group compared to the MASH-HMF group (Supplementary Fig. S1C) (*p* = 0.0030). However, spleen weight did not differ significantly between the two groups (Supplementary Fig. S1D) (*p* = 0.1096). Representative liver images again confirmed improved hepatic morphology in the FV-HMF group (Supplementary Fig. S1E).

### Alleviation of Liver Toxicity and Hepatic Lipid Accumulation by *F. venenatum*-Based Mycoprotein in HCF and HMF Diet-Fed Mice

**Fig. 2:**
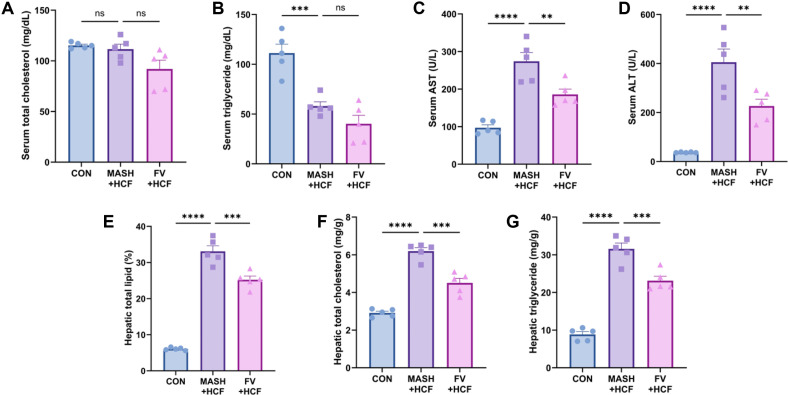
*F. venenatum* reduced liver toxicity and lipid accumulation in HCF diet-fed mice. (**A**) Serum total cholesterol. (**B**) Serum triglyceride. (**C**) Serum AST. (**D**) Serum ALT. (**E**) Hepatic total lipid. (**F**) Hepatic total cholesterol. (**G**) Hepatic triglyceride. Data are expressed as means ± SEM. Statistical analysis was performed using one-way ANOVA and differences were considered significant when *p*-value was below 0.05 (*), 0.01 (**), 0.001 (***), 0.0001 (****). CON group, received an oral administration of 200 μl of PBS along with a normal diet; MASH-HCF group, received an oral administration of 200 μL of PBS along with a HCF diet for MASH development; FV-HCF group, received an oral administration of *F. venenatum* at a dose of 800 mg/kg, suspended in 200 μL of PBS, along with a HCF diet for MASH development.

At week 3, serum samples were analyzed to evaluate systemic lipid profiles and markers of hepatic toxicity. In the HCF model, serum total cholesterol and triglyceride levels showed a decreasing trend in the FV-HCF group compared to the MASH-HCF group, however, the differences did not reach statistical significance (Fig. 2A and 2B) (*p* = 0.0818 for serum total cholesterol level and *p* = 0.2593 for serum triglyceride level). Notably, serum AST levels were significantly reduced in the FV-HCF group relative to the MASH-HCF group (Fig. 2C) (*p* = 0.0068) with a similar reduction observed in serum ALT levels (Fig. 2D) (*p* = 0.0091). To quantify hepatic lipid accumulation, total lipids were extracted from liver tissues. The FV-HCF group exhibited a significant reduction in total hepatic lipid content compared to the MASH-HCF group (Fig. 2E) (*p* = 0.0007). Consistently, hepatic total cholesterol and triglyceride levels were markedly lower in the FV-HCF group (Fig. 2F and 2G) (*p* = 0.0001 and *p* = 0.0009, respectively). Consistent results were observed in HMF-diet mice model. Serum total cholesterol and triglyceride levels did not differ significantly between the MASH-HMF and FV-HMF groups (Supplementary Fig. S2A and 2B) (*p* = 0.1244 and *p* = 0.0710, respectively). However, serum AST and ALT levels were significantly decreased in the FV-HMF group (Supplementary Fig. S2C) (*p* = 0.0054 and *p* = 0.0094, respectively). Total lipids were extracted to assess hepatic lipid accumulation. Hepatic total lipid content was significantly reduced in the FV-HMF group compared to the MASH-HMF group (Supplementary Fig. S2E) (*p* = 0.0056). Consistently, hepatic total cholesterol and triglyceride levels were also significantly lower in the FV-HMF group than MASH-HMF group (Fig. 2F and 2G) (*p* = 0.0050 for hepatic total cholesterol level and *p* = 0.0015 for hepatic total triglyceride level).

### Amelioration of Hepatic Steatosis and Fibrosis by *F. venenatum*-Based Mycoprotein in HCF and HMF Diet-Fed Mice

**Fig. 3:**
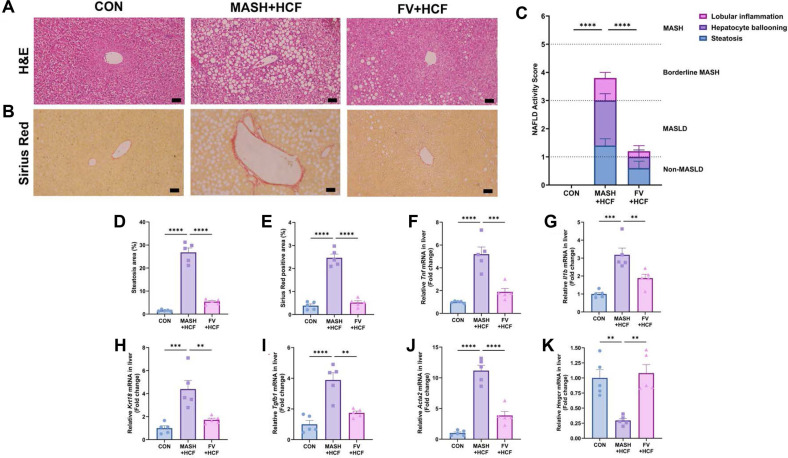
*F. venenatum* ameliorated hepatic steatosis and fibrosis in HCF diet-fed mice. (**A**) Representative images of hematoxylin and eosin-stained liver sections. Scale bar, 50 μm. (**B**) Representative images of Sirius Red-stained liver sections. Scale bar, 50 μm. (**C**) Nonalcoholic fatty liver disease activity score (NAS). (**D**) Hepatic steatosis area. (**E**) Hepatic fibrotic area. (**F-K**) Hepatic mRNA expression levels of genes associated with MASH progression. Inflammatory cytokines: (**F**) *Tnf* and (**G**) *Il1b*. Fibrosis-related genes: (**H**) *Krt18*, (**I**) *Tgfb1*, and (**J**) *Acta2*. Cholesterol metabolism-related genes: (**K**) *Hmgcr*. Gene expression was measured by qRT-PCR and normalized to the housekeeping gene *Hprt1*. Data are expressed as means ± SEM. Statistical analysis was performed using one-way ANOVA and differences were considered significant when *p*-value was below 0.05 (*), 0.01 (**), 0.001 (***), 0.0001 (****). CON group, received an oral administration of 200 μl of PBS along with a normal diet; MASH-HCF group, received an oral administration of 200 μL of PBS along with a HCF diet for MASH development; FV-HCF group, received an oral administration of *F. venenatum* at a dose of 800 mg/kg, suspended in 200 μL of PBS, along with a HCF diet for MASH development.

To evaluate the impact of *F. venenatum* on hepatic lipid accumulation, and fibrosis, histological analyses were performed using H&E staining and Sirius Red staining. In both the HCF and HMF diet models, H&E staining revealed severe hepatic steatosis in the MASH group, whereas hepatic fat accumulation was significantly reduced in the FV group compared to the MASH group (Fig. 3A and 3D; Supplementary Fig. S3A and S3D) (*p* < 0.0001 for both comparisons). Sirius Red staining showed a substantial increase in fibrotic area in the MASH group, consistent with advanced histopathological features of MASH progression (Fig. 3B and 3E; Supplementary Fig. S3B and 3E) (both *p* < 0.0001). In contrast, *F. venenatum* supplementation led to a significant reduction in hepatic fibrosis in both dietary models (Fig. 3B and 3E) (*p* < 0.0001). Furthermore, NAS, which evaluates steatosis, hepatocyte ballooning, and lobular inflammation, was significantly decreased in the FV group compared to the corresponding MASH groups (Fig. 3C; Supplementary Fig. S3C) (*p* < 0.0001 for both).

### Regulation of Hepatic Gene Expression by *F. venenatum*-Based Mycoprotein in HCF and HMF Diet-Fed Mice

To assess the impact of *F. venenatum*-based mycoprotein on hepatic gene regulation, transcriptional profiling was performed by qRT-PCR. In the HCF diet model, hepatic mRNA levels of pro-inflammatory cytokines *Tnf* and *Il1b* were significantly reduced in the FV-HCF group relative to the MASH-HCF group (Fig. 3F and 3G) (*p* = 0.0002 for *Tnf* and *p* = 0.0091 for *Il1b*). The expression of *Krt18*, a well-known biomarker of hepatocellular injury and ballooning, was markedly downregulated in the FV-HCF group compared to the MASH-HCF group (Fig. 3H) (*p* = 0.0039). In addition, fibrosis-associated genes *Tgfb1* and *Acta2* exhibited significant suppression in the FV-HCF group compared to the MASH-HCF group (Fig. 3I and 3J) (*p* = 0.0012 for *Tgfb1* and *p* < 0.0001 for *Acta2*). Interestingly, *Hmgcr*, a key enzyme in cholesterol biosynthesis, was significantly upregulated in the FV-HCF group (Fig. 3K) (*p* = 0.0012). Consistent with these findings, mice fed the HMF diet exhibited similar gene expression trends. The FV-HMF group showed a significant reduction in *Tnf* and *Il1b* expression compared to the MASH-HMF group (Supplementary Fig. S3F and S3G) (*p* = 0.0181 for *Tnf* and *p* < 0.0001 for *Il1b*). In addition, the expression levels of *Krt18*, *Tgfb1*, and *Acta2* were significantly lower in the FV-HMF group (Supplementary Fig. S3H-3J) (*p* = 0.0005 for *Krt18*, *p* = 0.0005 for *Tgfb1*, and *p* < 0.0001 for *Acta2*), while *Hmgcr* expression was significantly elevated (Supplementary Fig. S3K) (*p* = 0.0372).

### Regulation of Intestinal Gene Expression by *F. venenatum*-Based Mycoprotein in HCF and HMF Diet-Fed Mice

**Fig. 4:**
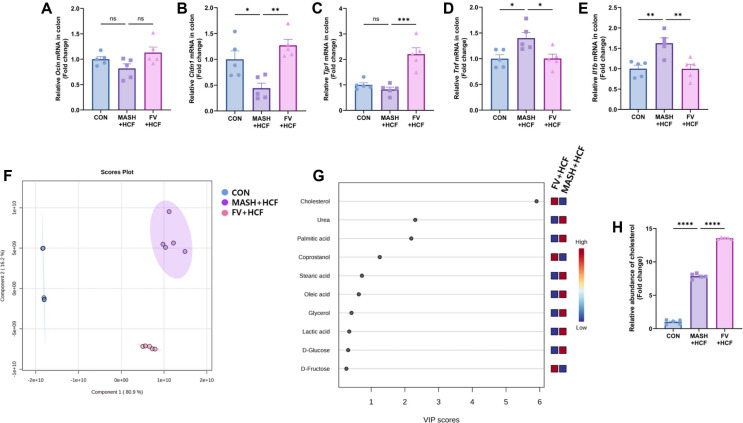
*F. venenatum* enhanced intestinal barrier integrity and cholesterol excretion in HCF diet-fed mice. (**A-E**) Intestinal mRNA expression levels of genes related to barrier integrity and inflammation. Barrier integrity-related genes: (**A**) *Ocln*, (**B**) *Cldn1*, and (**C**) *Tjp1*. Inflammatory cytokines: (**D**) *Tnf* and (**E**) *Il1b*. Gene expression was measured by qRT-PCR and normalized to the housekeeping gene *Hprt1*. (**F**) PLS-DA analysis of metabolites. (**G**) VIP score of PLS-DA analysis. (**H**) Relative abundance of cholesterol in feces. Data are expressed as means ± SEM. Statistical analysis was performed using one-way ANOVA and differences were considered significant when *p*-value was below 0.05 (*), 0.01 (**), 0.001 (***), 0.0001 (****). CON group, received an oral administration of 200 μl of PBS along with a normal diet; MASH-HCF group, received an oral administration of 200 μL of PBS along with a HCF diet for MASH development; FV-HCF group, received an oral administration of *F. venenatum* at a dose of 800 mg/kg, suspended in 200 μL of PBS, along with a HCF diet for MASH development. PLS-DA, partial least squares discriminant analysis; VIP, variable importance in projection.

Intestinal gene expression was analyzed to examine the effects of *F. venenatum* on intestinal barrier integrity and inflammation. The expression of *Ocln*, encoding occludin, a key component of tight junctions, did not differ significantly between the MASH-HCF and FV-HCF groups (Fig. 4A) (*p* = 0.0603). However, *Cldn1* expression (encoding claudin-1), was significantly upregulated in the FV group compared to the MASH-HCF group (Fig. 4B) (*p* = 0.0017). Similarly, *Tjp1*, which encodes the tight junction protein ZO-1, was also significantly increased in the FV-HCF group compared to the MASH-HCF group (Fig. 4C) (*p* = 0.0002). In contrast, the expression levels of *Tnf* and *Il1b*, pro-inflammatory cytokines involved in intestinal immune responses, were significantly downregulated in the FV-HCF group compared to the MASH-HCF group (Fig. 4D and 4E) (*p* = 0.0265 for *Tnf* and *p* = 0.0044 for *Il1b*). Collaborating with these findings, in the HMF-fed mice, the FV-HMF group showed significantly increased expression of *Ocln*, *Cldn1*, and *Tjp1* compared to the MASH-HMF group (Supplementary Fig. S4A-4C) (*p* = 0.0003 for *Ocln*, *p* = 0.0045 for *Cldn1*, and *p* = 0.0210 for *Tjp1*). In parallel, *Tnf* and *Il1b* levels were significantly suppressed in the FV-HMF group (Supplementary Fig. S4D and S4E)(*p* = 0.0169 for *Tnf* and *p* = 0.0116 for *Il1b*).

### Increased Excretion of Fecal Cholesterol by *F. venenatum*-based Mycoprotein in HCF and HMF Diet-Fed Mice

To assess the impact of *F. venenatum* supplementation on gut metabolic profiles, metabolomic analysis was performed using fecal samples. Partial least squares discriminant analysis (PLS-DA) revealed clear separation among the CON, MASH, and FV groups under both HCF and HMF dietary conditions, indicating distinct shifts in fecal metabolite composition (Fig. 4F; Supplementary Fig. S4F). Variable importance in projection (VIP) analysis identified ten key metabolites that discriminated between the FV and MASH groups (Fig. 4G; Supplementary Fig. S4G). Among these, cholesterol emerged as the top discriminant metabolite, showing significantly higher levels in the feces of the FV group compared to the MASH group under both diet models (Fig. 4g; Supplementary Fig. S4G). To confirm this observation, the relative abundance of fecal cholesterol was measured. Cholesterol levels were significantly elevated in the FV group compared to the MASH group in both HCF and HMF diet-fed mic (Fig. 4H; Supplementary Fig. S4H) (*p* = 0.0012 and *p* < 0.0001 in HCF and HMF diets, respectively).

### Modulation of Gut Microbiota by *F. venenatum*-based Mycoprotein in HCF and HMF Diet-Fed Mice

**Fig. 5:**
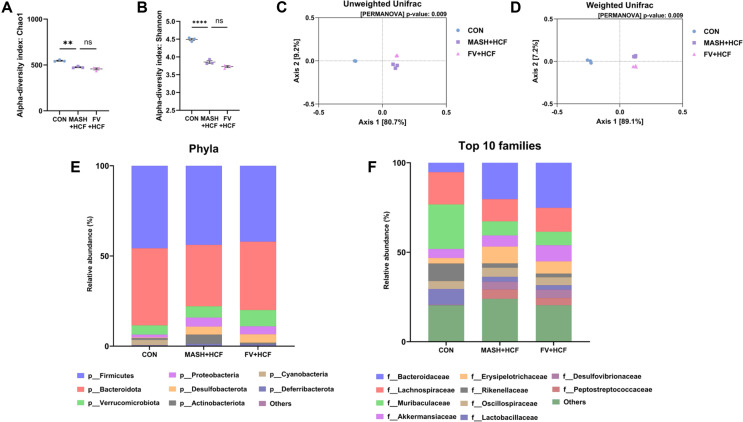
*F. venenatum* modulated gut microbiota in HCF diet-fed mice. (**A**) Comparison of alpha diversity based on Chao1 index values. (**B**) Comparison of alpha diversity based on Shannon index values. (**C**) Beta diversity analysis using unweighted UniFrac distances. (**D**) Beta diversity analysis using weighted UniFrac distances. (**E**) Relative abundance of microbial taxa at the phylum level. (**F**) Relative abundance of microbial taxa at the family level using the top 10 taxa. Statistical analysis was performed using one-way ANOVA and differences were considered significant when *p*-value was below 0.05 (*), 0.01 (**), 0.001 (***), 0.0001 (****). CON group, received an oral administration of 200 μL of PBS along with a normal diet; MASH-HCF group, received an oral administration of 200 μL of PBS along with a HCF diet for MASH development; FV-HCF group, received an oral administration of *F. venenatum* at a dose of 800 mg/kg, suspended in 200 μL of PBS, along with a HCF diet for MASH development.

**Fig. 6:**
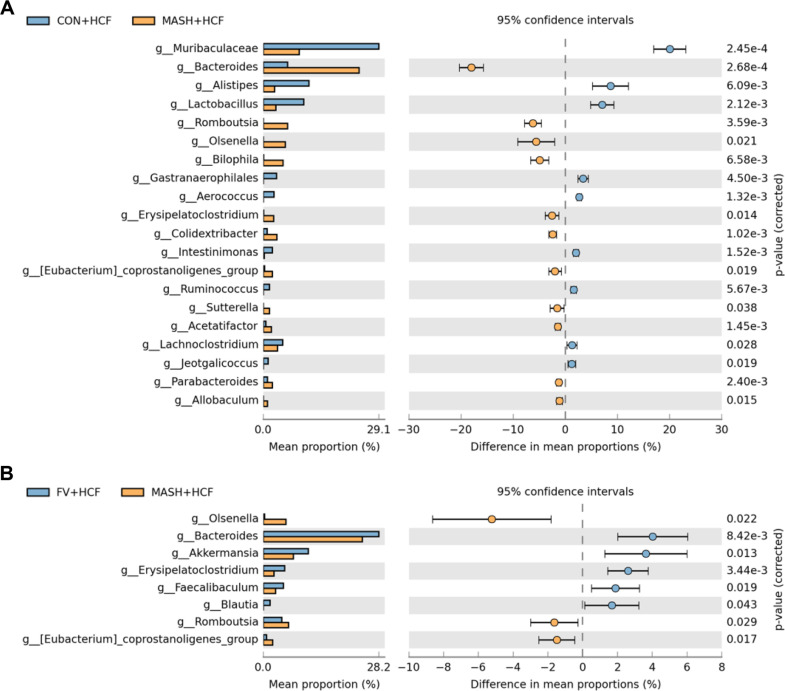
*F. venenatum* altered specific gut microbial composition in HCF diet-fed mice. (**A**) Differentially abundant genera between CON and MASH groups, identified using STAMP with Welch’s t-test (*p* < 0.05). (**B**) Differentially abundant genera between FV and MASH groups, identified using STAMP with Welch’s t-test (*p* < 0.05). Statistical analysis was performed using Welch’s t-test. CON group, received an oral administration of 200 μl of PBS along with a normal diet; MASH-HCF group, received an oral administration of 200 μL of PBS along with a HCF diet for MASH development; FV-HCF group, received an oral administration of *F. venenatum* at a dose of 800 mg/kg, suspended in 200 μL of PBS, along with a HCF diet for MASH development.

Microbiota profiling based on 16S rRNA sequencing was performed using fecal samples to evaluate the impact of *F. venenatum* supplementation on gut microbiota composition. Initially, in HCF diets experiment, alpha diversity was evaluated using the Chao1 and Shannon indices. The Chao1 index, reflecting species richness, showed no significant difference between the MASH-HCF and FV-HCF groups (Fig. 5A) (*p* = 0.2929). Similarly, the Shannon index, which accounts for both richness and evenness, revealed no statistical difference between the groups (Fig. 5B) (*p* = 0.0760). However, beta diversity analysis using both unweighted and weighted UniFrac distances revealed distinct clustering patterns among the CON, MASH-HCF, and FV-HCF groups (Fig. 5C and 5D). At the phylum level, Verrucomicrobiota exhibited the highest relative abundance in the FV-HCF group whereas Proteobacteria and Deferribacterota were markedly enriched in the MASH-HCF group (Fig. 5E). At the family level, *Akkermansia*ceae was more prominent in the FV-HCF group, while *Erysipelotrichaceae* and *Peptostreptococcaceae* showed the highest abundance in the MASH-HCF group (Fig. 5F). Genus-level comparisons between the CON and MASH-HCF groups revealed a significant reduction in *Lactobacillus* and a significant increase in *Romboutsia* in the MASH-HCF group (Fig. 6A) (*p* = 0.0021 for *Lactobacillus* and *p* = 0.0036 for *Romboutsia*). When comparing the MASH-HCF and FV-HCF groups, *Akkermansia* was significantly elevated in the FV-HCF group (*p* = 0.013), whereas *Olsenella* and *Romboutsia* were significantly reduced (*p* = 0.022 and *p* = 0.029, respectively; Fig. 6B).

On the other hand, under the HMF diet conditions, alpha diversity metrics showed no significant differences between the MASH-HMF and FV-HMF groups (Supplementary Fig. S5A and 5B) (*p* = 0.6768 for Chao1 and *p* = 0.8362 for Shannon). Nonetheless, beta diversity analysis revealed distinct microbial clustering among the CON, MASH-HMF, and FV-HMF groups (Supplementary Fig. S5C and 5D). Consistent with results from HCF model, at the phylum level, Verrucomicrobiota was most abundant in the FV-HMF group, whereas Proteobacteria and Deferribacterota were enriched in the MASH-HMF group (Supplementary Fig. S5E). At the family level, *Morganellaceae* and *Tannerellaceae* exhibited the highest relative abundance in the MASH-HMF group compared to the other groups (Supplementary Fig. S5F). At the genus level, comparison between the CON and MASH-HMF groups revealed a significant reduction in *Lactobacillus* abundance in the MASH-HMF group, whereas *Romboutsia* was significantly increased (Supplementary Fig. S6A) (*p* = 0.0014 for *Lactobacillus* and *p* = 0.026 for *Romboutsia*). Comparison between the MASH-HMF and FV-HMF groups revealed that *Akkermansia* abundance was significantly elevated in the FV-HMF group compared to the MASH-HMF group (Supplementary Fig. S6B) (*p* = 0.042).

## Discussion

In this study, we demonstrated that dietary supplementation with *F. venenatum*-derived mycoprotein exerted protective effects against the progression of MASH. Two independent dietary models were employed: MASH was induced using a high-fat, high-fructose, choline-deficient (HCF) diet in the first experiment, and a more severe phenotype was elicited in the second experiment using a diet additionally deficient in methionine (HMF). Despite differences in disease severity, *F. venenatum*-based mycoprotein consistently attenuated hallmark pathological features of MASH, including hepatic steatosis, fibrosis, and inflammation. Furthermore, *F. venenatum*-based mycoprotein improved intestinal barrier function, increased the abundance of beneficial bacterial taxa, and decreased levels of potentially pathogenic bacteria, supporting its potential as a functional dietary strategy to prevent MASH progression.

In both HCF and HMF experiments, *F. venenatum*-based mycoprotein significantly alleviated hepatic steatosis and fibrosis, accompanied by reduced hepatic expression of *Tnf* and *Il1b*, suggesting that its hepatoprotective effects are mediated through the suppression of hepatic inflammation. TNF-α and IL-1β, pro-inflammatory cytokines primarily produced by Kupffer cells, play central roles in MASH pathogenesis by amplifying inflammatory signaling, promoting hepatocyte injury, and stimulating fibrogenic responses via activation of hepatic immune cells and the inflammasome pathway [35]. Chronic hepatic inflammation not only drives fibrogenesis but also exacerbates lipid accumulation, further contributing to MASH progression [24, 36, 37]. Inflammatory persistence may exacerbate tissue damage and promote fibrosis through abnormal wound healing responses, contributing to the progression of MASH [38, 39]. The observed downregulation in *Tnf* and *Il1b*, along with decreased hepatic lipid accumulation, suggest that *F. venenatum*-based mycoprotein may mitigate MASH progression by modulating inflammatory signaling.

Excessive dietary cholesterol intake is associated with increased risk and severity of MASLD and can accelerate MASH progression by triggering hepatic inflammatory responses [38, 40]. In the present study, fecal metabolomics results revealed that *F. venenatum* supplementation increased fecal cholesterol excretion, implying reduced intestinal cholesterol absorption. This finding is consistent with previous reports on *F. venenatum* [29]. A similar mechanism of MASH improvement via reduced intestinal cholesterol absorption has been demonstrated using ezetimibe. Ezetimibe inhibits the Niemann-Pick C1-Like 1 (NPC1L1) transporter on the intestinal epithelium, blocking dietary cholesterol uptake [41]. This mechanism ultimately reduces hepatic cholesterol accumulation, which contributes to the prevention of lipotoxicity, inflammation, and fibrogenesis in the liver [41-44]. Supporting this concept, dietary cholesterol removal from a high-fat, high-cholesterol diet has been shown to prevent hepatic inflammation and Kupffer cell activation in mice [45]. Hepatic *Hmgcr* expression was significantly upregulated in the FV group compared with the MASH group. Although this increase could theoretically reflect enhanced cholesterol synthesis, it is more plausibly explained as a compensatory response to reduced intestinal cholesterol absorption, given the marked rise in fecal cholesterol excretion [29]. Therefore, the increased fecal cholesterol excretion by *F. venenatum*-based mycoprotein suggests that a physiological reduction in intestinal cholesterol uptake may attenuate MASH progression.

Supplementation of *F. venenatum*-based mycoprotein enhanced intestinal barrier integrity, as evidenced by the upregulation of tight junction-associated genes in both HCF and HMF model. Increased expression of these genes is a well-established marker of improved barrier function [46-49]. Gut barrier dysfunction is a well-established contributor to MASH pathogenesis, facilitating translocation of bacterial components such as LPS, which in turn activates hepatic inflammation via portal circulation [21, 38]. Supporting this mechanism, previous studies have demonstrated that mice lacking junctional adhesion molecule A exhibit enhanced intestinal permeability and bacterial translocation, exacerbating MASH pathology [50]. In addition, *F. venenatum*-based mycoprotein reduced the gene expression of pro-inflammatory cytokines *Tnf* and *Il1b* in the colon, suggesting its potential to suppress intestinal inflammation. This is consistent with previous findings showing that gut-targeted anti-inflammatory agents in high-fat diet-fed mice alleviate intestinal inflammation, improve gut barrier integrity, and attenuate hepatic steatosis [51]. Taken together, these results suggest that *F. venenatum*-based mycoprotein may protect against MASH progression in part by reinforcing gut barrier function, which may help limit the translocation of endotoxins such as LPS from the intestine to the liver. These improvements in intestinal barrier integrity may be partly explained by the alterations in gut microbiota composition observed in this study.

Fecal metagenomic analysis revealed that at the phylum level in both HCF and HMF experiments, Proteobacteria and Deferribacterota, both commonly associated with dysbiosis and increased LPS biosynthesis, were enriched in MASH-induced mice and reduced following *F. venenatum* treatment. Proteobacteria are a well-recognized marker of gut dysbiosis, and their abundance has been reported to be increased in MASH compared with healthy controls [52-54]. Similarly, feeding high fructose to mice has been shown to increase Proteobacteria abundance, leading to elevated LPS levels and promoting hepatic steatosis [55]. Deferribacterota has also been reported to be higher in MASH conditions [56]. In contrast, the abundance of Verrucomicrobiota, a phylum generally considered beneficial, was most abundant in the FV group. Consistent with this finding, previous studies have reported that elevated Verrucomicrobiota levels have been associated with improved metabolic parameters and MASH alleviation [57-59].

At the genus level in both HCF and HMF models, *Lactobacillus* was significantly reduced in the MASH group compared with the CON group, whereas *Romboutsia* was significantly increased in the MASH group. *Lactobacillus* is widely recognized as a beneficial bacterium that produces lactic acid, enhances intestinal barrier function, and supports host metabolic health [60-64]. In contrast, *Romboutsia* was significantly enriched in the MASH group, in line with earlier studies reporting its association with obesity and MASLD [65-68]. Moreover, a previous study revealed that *Romboutsia* shows a positive correlation with TNF-α levels in both serum and liver [69]. Furthermore, human studies have reported associations between *Romboutsia* and hepatocellular carcinoma. In fecal samples from patients with hepatocellular carcinoma, *Romboutsia* was significantly enriched compared with healthy controls and demonstrated strong diagnostic potential, highlighting its value as a promising non-invasive biomarker for hepatocellular carcinoma detection [70, 71].

*Akkermansia*, the dominant genus within *Verrucomicrobiota*, was significantly enriched in FV group compared to MASH group. *Akkermansia muciniphila* is a mucin-degrading bacterium with established probiotic properties, including modulation of host metabolism, immunoregulation, and enhancement of mucosal barrier function [72, 73]. Located in the mucus layer, *Akkermansia* supports mucin turnover and goblet cell function, facilitating epithelial renewal and barrier integrity [74]. This increase in *Akkermansia* abundance is consistent with previous studies reporting its marked depletion during MASH progression [75]. An increase in *Akkermansia* abundance has been associated with improvements in MASH and dihydromyricetin treatment in MASH-induced mice attenuated disease progression while increasing *Akkermansia* abundance [58]. Supplementation with *Akkermansia* in MASLD models reduced body fat, alleviated hepatic steatosis, lowered pro-inflammatory cytokine levels, and improved gut barrier integrity by upregulating tight junction protein expression [76, 77]. Similarly, in a high-fat diet-induced MASLD mouse model, *A. muciniphila* administration upregulated the mRNA expression of intestinal tight junction proteins and reduced serum LPS levels [78]. Supplementation of *F. venenatum*-based mycoprotein led to a concurrent reduction in harmful bacteria, enrichment of beneficial bacteria, decreased expression of pro-inflammatory cytokines, and upregulation of gut barrier-related genes, suggesting a coordinated mechanism linking gut microbial modulation to improved intestinal integrity. Specifically, the decrease in LPS-associated bacteria, such as Proteobacteria and Deferribacterota, likely reduces the luminal endotoxin burden, while the enrichment of beneficial microbes, including *Akkermansia*, may contribute to improved epithelial barrier function [79, 80]. Strengthening of the intestinal barrier would consequently limit the translocation of endotoxins such as LPS into the portal circulation, thereby attenuating hepatic inflammatory signaling [81, 82]. This mechanistic sequence provides a mechanistic basis for the protective effects of *F. venenatum*-based mycoprotein against MASH.

Several limitations of this study should be acknowledged. First, as this study was conducted in a mouse model, the findings should be interpreted with caution when extrapolating to human metabolic disease due to differences between mice and humans. Second, the intervention period was relatively short, and the long-term effects of *F. venenatum* supplementation on disease progression remain unclear. Third, although the HCF and HMF diets effectively induced MASH phenotypes, they may not fully recapitulate the complex metabolic features observed in human disease under Western-style dietary conditions. Fourth, although our findings suggest that *F. venenatum* may protect against MASH by strengthening the gut barrier and reducing endotoxin translocation, direct measurements of circulating or hepatic endotoxin levels are required to substantiate this mechanism. Future studies employing fecal microbiota transplantation or germ-free mouse models are warranted to establish causal relationships between microbial shifts and disease amelioration.

## Conclusion

**Fig. 7:**
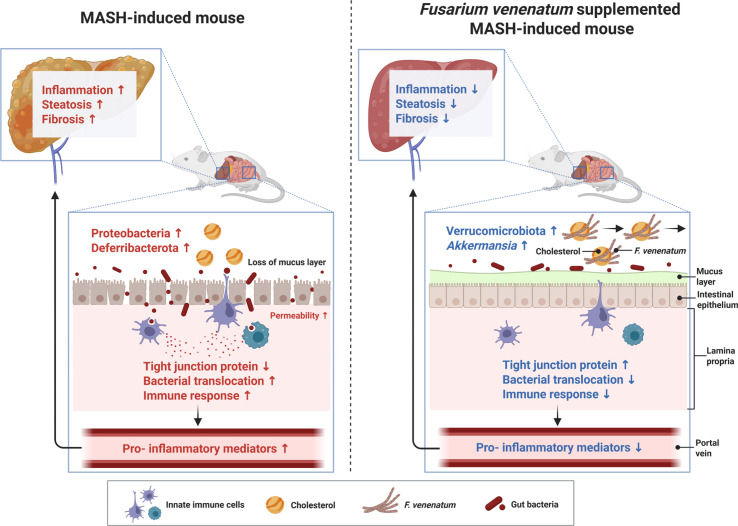
Schematic overview of the mechanism underlying the protective effects of *F. venenatum* in a MASH-induced mouse model, as revealed by integrated multi-omics analysis. Feeding mice a MASH-inducing diet increased the intestinal abundance of potentially harmful taxa such as Proteobacteria and Deferribacterota, accompanied by reduced expression of tight junction proteins and activation of intestinal immune responses. These alterations may have facilitated bacterial translocation and the entry of gut-derived endotoxins such as LPS into the portal circulation, contributing to hepatic inflammation, increased steatosis, and fibrogenic activation, ultimately driving MASH development. In contrast, dietary supplementation with *F. venenatum* reshaped the gut microbiota toward a more favorable composition, increasing beneficial taxa such as Verrucomicrobiota and *Akkermansia*. It also enhanced the expression of tight junction proteins and attenuated intestinal inflammatory responses, changes that may collectively help limit bacterial translocation and thereby ameliorate MASH pathology. Additionally, *F. venenatum* increased fecal cholesterol excretion, suggesting reduced intestinal cholesterol absorption. This image was created using BioRender.

In conclusion, dietary supplementation with *F. venenatum*-derived mycoprotein effectively protected against the development and progression of MASH in both HCF and HMF diet-induced models (Fig. 7). Rather than being driven by a single pathway, these protective effects are consistent with coordinated modulation of the gut-liver axis, including improved intestinal barrier integrity, reduced inflammatory signaling within the gut and liver, decreased intestinal cholesterol absorption, and altered gut microbial composition. Together, these interconnected changes provide a mechanistic basis for the observed attenuation of hepatic steatosis, fibrogenesis, and inflammation. Taken together, these findings highlight the potential of *F. venenatum*-based mycoprotein as a promising functional dietary strategy for the prevention and management of MASH by modulating the gut-liver axis.

## Supplemental Materials

Supplementary data for this paper are available on-line only at http://jmb.or.kr.



## Figures and Tables

**Table 1:** Composition of diets used in this study.

	Normal diet	HCF diet	HMF diet
**Ingredients, g/kg**			
Sucrose	94.8	64.8	64.8
Fructose	0.0	100.0	100.0
Maltodextrin	93.4	90.3	93.2
Corn starch	361.9	0.0	0.0
Cellulose	65.1	65.1	65.1
Soybean oil	64.1	32.6	32.6
Tallow	0.0	319.1	319.1
Glutamate	49.7	49.7	49.7
Glycine	3.9	3.9	3.9
Lysine	17.2	17.2	17.2
Arginine	7.8	7.8	7.8
Leucine	20.6	20.6	20.6
Threonine	9.4	9.4	9.4
Isoleucine	9.9	9.9	9.9
Valine	12.1	12.1	12.1
Phenylalanine	10.9	10.9	10.9
Tyrosine	12.0	12.0	12.0
Histidine	6.0	6.0	6.0
Alanine	6.6	6.6	6.6
Aspartate	15.8	15.8	15.8
Cystine	5.5	5.5	5.5
Proline	23.2	23.2	23.2
Serine	13.0	13.0	13.0
Tryptophan	2.7	2.7	2.7
Methionine	3.9	3.9	1.0
Sodium bicarbonate	9.8	9.8	9.8
Mineral mix (AIN-76)	65.1	65.1	65.1
Vitamin mix (AIN-76A)	13.0	13.0	13.0
Choline bitartrate	2.6	0.0	0.0
Cholesterol	0.0	10.0	10.0
Total	1000.0	1000.0	1000.0
**Calculated composition**			
ME, kcal/kg	2289.1	3652.6	3652.6
Carbohydrate, g/kg	500.0	248.6	251.4
Crude protein, g/kg	182.8	182.8	181.1
Crude fat, g/kg	64.4	351.9	351.9

**Table 2:** Primer sequences used in this study.

Genes	Sequence
*Krt18*	F: 5'- CAAGTACTGGTCTCAGCAGATTGA -3'
	R: 5'- CTTGGTGGTGACAACTGTGGTA -3'
*Tgfb1*	F: 5'- GTCACTGGAGTTGTACGGCA -3'
	R: 5'- GGGCTGATCCCGTTGATTTC -3'
*Acta2*	F: 5'- CCAGCCATCTTTCATTGGGATG -3'
	R: 5'- TACCCCCTGACAGGACGTTG -3'
*Hmgcr*	F: 5'- AGCTTGCCCGAATTGTATGTG -3'
	R: 5'- TCTGTTGTGAACCATGTGACTTC -3'
*Tnf*	F: 5'- AGGGTCTGGGCCATAGAACT -3'
	R: 5'- CCACCACGCTCTTCTGTCTAC -3'
*Il1b*	F: 5'- CTGAACTCAACTGTGAAATGC -3'
	R: 5'- TGATGTGCTGCTGCGAGA -3'
*Ocln*	F: 5'- TCACTTTTCCTGCGGTGACT -3'
	R: 5'- GGGAACGTGGCCGATATAATG -3'
*Cldn1*	F: 5'- CCTTCGGGAGCTCAGGTGCG -3'
	R: 5'- CCGCGTTGGCCATGACTCT -3'
*Tjp1*	F: 5'- GCTGCCTGAACCTCTACTC -3'
	R: 5'- TTGCTCATAACTTCGCGGGT -3'
*Hprt1*	F: 5'- TCAGTCAACGGGGGACATAAA -3'
	R: 5'- GGGGCTGTACTGCTTAACCAG -3'

## References

[1] Teng ML, Ng CH, Huang DQ, Chan KE, Tan DJ, Lim WH (2022). Global incidence and prevalence of nonalcoholic fatty liver disease. Clin. Mol. Hepatol..

[2] Yilmaz Y. 2023. Presented at the Hepatology Forum.

[3] Lee HL, Kim JM, Go MJ, Lee HS, Kim JH, Kim IY (2025). Fermented *Protaetia brevitarsis* larvae alleviates high-fat diet-induced non-alcoholic fatty liver disease in C57BL/6 mice via regulation of lipid accumulation and inflammation. J. Microbiol. Biotechnol..

[4] Younossi Z, Anstee QM, Marietti M, Hardy T, Henry L, Eslam M (2018). Global burden of NAFLD and NASH: trends, predictions, risk factors and prevention. Nat. Rev. Gastroenterol. Hepatol..

[5] Hsu M, Karkossa I, Schäfer I (2020). Mitochondrial transfer by human mesenchymal stromal cells ameliorates hepatocyte lipid load in a mouse model of NASH. Biomedicines.

[6] Zhu K, Xiang J, Lu F, Wang J, Zhang Y, Feng H (2025). The therapeutic effect and mechanism of *Lactobacillus gardneri* on nonalcoholic fatty liver disease. J. Microbiol. Biotechnol..

[7] Mun D, Ryu S, Lee DJ, Kwak M-J, Choi H, Kang AN (2025). Bovine colostrum-derived extracellular vesicles protect against non-alcoholic steatohepatitis by modulating gut microbiota and enhancing gut barrier function. Curr. Res. Food Sci..

[8] Hashimoto E, Yatsuji S, Kaneda H, Yoshioka Y, Taniai M, Tokushige K (2005). The characteristics and natural history of Japanese patients with nonalcoholic fatty liver disease. Hepatol. Res..

[9] Lee D, Goh TW, Kang MG, Choi HJ, Yeo SY, Yang J (2022). Perspectives and advances in probiotics and the gut microbiome in companion animals. J. Anim. Sci. Technol..

[10] Kang M, Yun B, Mun D, Kim S, Jeong KC, Kim Y (2025). Bovine colostrum-derived exosomes alleviate muscle degeneration by modulating gut microbiota and metabolic homeostasis in atrophy models. J. Anim. Sci. Technol..

[11] Lee JH, Kim S, Kim ES, Keum GB, Doo H, Kwak J (2023). Comparative analysis of the pig gut microbiome associated with the pig growth performance. J. Anim. Sci. Technol..

[12] Jeon K, Lee J, Song M, Park K, Chang S, Song D (2025). Alternative nutritional evaluation of black soldier fly with various substrates in cat diets and its effects on fecal microbiota. J. Anim. Sci. Technol..

[13] Choi Y, Kang A, Kang M-G, Lee DJ, Kyoung D, Kwak M-J, *et al*. 2025. Exploring synergistic effect of bacteriophages with probiotics against multidrug resistant *Salmonella* Typhimurium in a simulated chicken gastrointestinal system using metagenomic-and culturomic approaches. *J. Anim. Sci. Technol. *https://doi.org/10.5187/jast.2025.e30. 10.5187/jast.2025.e30

[14] Choi Y, Kang A, Seo E, Lee DJ, Park J, Kim Y (2026). Combination of bacteriophage-probiotics alleviates intestinal barrier dysfunction by regulating gut microbiome in a chick model of multidrug-resistant *Salmonella* infection. J. Anim. Sci. Biotechnol..

[15] Han H, Jiang Y, Wang M, Melaku M, Liu L, Zhao Y (2023). Intestinal dysbiosis in nonalcoholic fatty liver disease (NAFLD): Focusing on the gut-liver axis. Crit. Rev. Food Sci. Nutr..

[16] Hsu CL, Schnabl B (2023). The gut-liver axis and gut microbiota in health and liver disease. Nat. Rev. Microbiol..

[17] Choi S, Kim JW, Ji J-H, Kim K-M, Park J-K, Han GH, *et al*. 2025. Anti-inflammatory effects of Canis familiaris gingival tissue-derived microorganisms on *Porphyromonas gingivalis*-derived lipopolysaccharide-treated RAW 264.7 macrophages. *J. Anim. Sci. Technol.* https://doi.org/10.5187/jast.2025.e38. 10.5187/jast.2025.e38 PMC13266149 42305229

[18] Wang J, Gao Y, Cheng C, Li Y, Zhang X, Yao D, *et al*. 2024. Dangguibuxue decoction protects against lipopolysaccharides-induced mastitis in bovine mammary epithelial cells in vitro. *J. Anim. Sci. Technol.* https://doi.org/10.5187/jast.2024.e63. 10.5187/jast.2024.e63 PMC13260098 42291113

[19] Paing YMM, Lee SH (2025). Protective effects of enzymatically digested velvet antler polypeptides on mitochondria in primary astrocytes. J. Anim. Sci. Technol..

[20] Park B, Kang D, Jang S, Kim U, Kim J, Choi B (2025). Effect of dietary natural phytoncide on blood characteristics to lipopolysaccharide challenge of Hanwoo cattle. J. Anim. Sci. Technol..

[21] Tripathi A, Debelius J, Brenner DA, Karin M, Loomba R, Schnabl B (2018). The gut-liver axis and the intersection with the microbiome. Nat. Rev. Gastroenterol. Hepatol..

[22] Milosevic I, Vujovic A, Barac A, Djelic M, Korac M, Radovanovic Spurnic A (2019). Gut-liver axis, gut microbiota, and its modulation in the management of liver diseases: a review of the literature. Int. J. Mol. Sci..

[23] Cho Y-C, Yao L, Li X, Yoo G, Choi SY, Cho N (2025). 8-Methoxybicolosin C from lespedeza bicolor attenuates inflammation and oxidative stress via Nrf2/HO-1 and NF-κB/MAPK pathways in lipopolysaccharide-induced mouse kupffer cells. J. Microbiol. Biotechnol..

[24] Song Q, Zhang X (2022). The role of gut-liver axis in gut microbiome dysbiosis associated NAFLD and NAFLD-HCC. Biomedicines.

[25] Konturek PC, Harsch IA, Konturek K, Schink M, Konturek T, Neurath MF (2018). Gut-liver axis: how do gut bacteria influence the liver?. Med. Sci..

[26] Aron-Wisnewsky J, Vigliotti C, Witjes J, Le P, Holleboom AG, Verheij J, *et al*. 2020. *Nat. Rev. Gastroenterol. Hepatol.* **17:** 279-297. https://doi.org/10.1038/s41575-020-0269-9. 10.1038/s41575-020-0269-9 32152478

[27] Finnigan TJ, Wall BT, Wilde PJ, Stephens FB, Taylor SL, Freedman MR (2019). Mycoprotein: the future of nutritious nonmeat protein, a symposium review. Curr. Dev. Nutr..

[28] Colosimo R, Mulet-Cabero A-I, Warren FJ, Edwards CH, Finnigan TJ, Wilde PJ (2020). Mycoprotein ingredient structure reduces lipolysis and binds bile salts during simulated gastrointestinal digestion. Food Funct..

[29] Lee DJ, Kang AN, Lee J, Kwak M-J, Mun D, Lee D (2024). Molecular characterization of *Fusarium venenatum*-based microbial protein in animal models of obesity using multi-omics analysis. Commun. Biol..

[30] Jacobson MF, DePorter J (2018). Self-reported adverse reactions associated with mycoprotein (Quorn-brand) containing foods. Ann. Allergy Asthma Immunol..

[31] Finnigan T. 2011. Mycoprotein: origins, production and properties. *Handbook of food proteins.* pp.335-352. 10.1533/9780857093639.335

[32] Wiebe M (2002). Myco-protein from *Fusarium venenatum*: a well-established product for human consumption. Appl. Microbiol. Biotechnol..

[33] Kleiner DE, Brunt EM, Van Natta M, Behling C, Contos MJ, Cummings OW (2005). Design and validation of a histological scoring system for nonalcoholic fatty liver disease. Hepatology.

[34] Brunt EM, Kleiner DE, Wilson LA, Belt P, Neuschwander‐Tetri BA, Network NCR (2011). Nonalcoholic fatty liver disease (NAFLD) activity score and the histopathologic diagnosis in NAFLD: distinct clinicopathologic meanings. Hepatology.

[35] Rafaqat S, Gluscevic S, Mercantepe F, Rafaqat S, Klisic A (2024). Interleukins: pathogenesis in non-alcoholic fatty liver disease. Metabolites.

[36] Tilg H, Moschen AR (2010). Evolution of inflammation in nonalcoholic fatty liver disease: the multiple parallel hits hypothesis. Hepatology.

[37] Machado MV, Diehl AM (2016). Pathogenesis of nonalcoholic steatohepatitis. Gastroenterology.

[38] Schuster S, Cabrera D, Arrese M, Feldstein AE (2018). Triggering and resolution of inflammation in NASH. Nat. Rev. Gastroenterol. Hepatol..

[39] Lee S-J, Yang J, Keum GB, Kwak J, Doo H, Choi S (2024). Therapeutic potential of *Lactiplantibacillus plantarum* FB091 in alleviating alcohol-induced liver disease through gut-liver axis. J. Microbiol. Biotechnol..

[40] Enjoji M, Yasutake K, Kohjima M, Nakamuta M (2012). Nutrition and nonalcoholic fatty liver disease: the significance of cholesterol. Int. J. Hepathol..

[41] Enjoji M, Machida K, Kohjima M, Kato M, Kotoh K, Matsunaga K (2010). NPC1L1 inhibitor ezetimibe is a reliable therapeutic agent for non-obese patients with nonalcoholic fatty liver disease. Lipids Health Dis..

[42] Musso G, Cassader M, Gambino R (2011). Cholesterol-lowering therapy for the treatment of nonalcoholic fatty liver disease: an update. Curr. Opin. Lipidol..

[43] Musso G (2014). Ezetimibe in the balance: can cholesterol-lowering drugs alone be an effective therapy for NAFLD?. Diabetologia.

[44] Van Rooyen DM, Gan LT, Yeh MM, Haigh WG, Larter CZ, Ioannou G (2013). Pharmacological cholesterol lowering reverses fibrotic NASH in obese, diabetic mice with metabolic syndrome. J. Hepatol..

[45] Wouters K, van Gorp PJ, Bieghs V, Gijbels MJ, Duimel H, Lütjohann D (2008). Dietary cholesterol, rather than liver steatosis, leads to hepatic inflammation in hyperlipidemic mouse models of nonalcoholic steatohepatitis. Hepatology.

[46] Preesong P, Lertwatcharasarakul P, Pongmanee K, Seemacharoensri A, Tactacan G, Chaosap C (2024). Effect of microencapsulated organic acids-essential oils blend and protease on performance and gut health of broilers under nutritional challenges. J. Anim. Sci. Technol..

[47] Han M-G, Lee R, Sim H-W, Lee W-Y, Park Y-B, Lee S-H, *et al*. 2025. Effects of *Bacillus licheniformis* and *Bacillus subtilis* on growth performance, gut health, and immunity in pigs. *J. Anim. Sci. Technol.* 10.5187/jast.2400475

[48] Keum GB, Doo H, Kwak J, Sun X, Cho J, Kim HB (2025). Effects of phytobiotics on intestinal barrier function and gut microbiome in weaned piglets challenged with enterotoxigenic *Escherichia coli*. J. Anim. Sci. Technol..

[49] Kyoung H, Park Y, Park KI, Ahn J, Kang Y, Hwang H, *et al*. 2026. Dietary supplement *Lactococcus lactis* improved anti-inflammatory responses and intestinal barrier function of weaned pigs. *J. Anim. Sci. Technol.* https://doi.org/10.5187/jast.2600048. 10.5187/jast.2600048

[50] Rahman K, Desai C, Iyer SS, Thorn NE, Kumar P, Liu Y (2016). Loss of junctional adhesion molecule a promotes severe steatohepatitis in mice on a diet high in saturated fat, fructose, and cholesterol. Gastroenterology.

[51] Luck H, Tsai S, Chung J, Clemente-Casares X, Ghazarian M, Revelo XS (2015). Regulation of obesity-related insulin resistance with gut anti-inflammatory agents. Cell Metab..

[52] Sobhonslidsuk A, Chanprasertyothin S, Pongrujikorn T, Kaewduang P, Promson K, Petraksa S (2018). The association of gut microbiota with nonalcoholic steatohepatitis in Thais. BioMed Res. Int..

[53] Albhaisi SA, Bajaj JS (2021). The influence of the microbiome on NAFLD and NASH. Clin. Liver Dis..

[54] Zhu L, Baker SS, Gill C, Liu W, Alkhouri R, Baker RD (2013). Characterization of gut microbiomes in nonalcoholic steatohepatitis (NASH) patients: a connection between endogenous alcohol and NASH. Hepatology.

[55] Vasques-Monteiro IML, Silva-Veiga FM, Miranda CS, de Andrade Gonçalves ÉCB, Daleprane JB, Souza-Mello V (2021). A rise in Proteobacteria is an indicator of gut-liver axis-mediated nonalcoholic fatty liver disease in high-fructose-fed adult mice. Nutr. Res..

[56] Zhang W, Cheng W, Li J, Huang Z, Lin H, Zhang W. 2024. New aspects characterizing non-obese NAFLD by the analysis of the intestinal flora and metabolites using a mouse model. *mSystems* **9:** e01027-01023. https://doi.org/10.1128/msystems.01027-23. 10.1128/msystems.01027-23 38421203 PMC10949483

[57] Dong X, Xiong Y-T, He T, Zheng C, Li J, Zhuang Y (2024). Protective effects of Nogo-B deficiency in NAFLD mice and its multiomics analysis of gut microbiology and metabolism. Genes Nutr..

[58] Miao X, Luo P, Liu J, Wang J, Chen Y (2023). Dihydromyricetin ameliorated nonalcoholic steatohepatitis in mice by regulating the composition of serous lipids, bile acids and ileal microflora. Lipids Health Dis..

[59] Zhu X, Cai J, Wang Y, Liu X, Chen X, Wang H (2023). A high-fat diet increases the characteristics of gut microbial composition and the intestinal damage associated with non-alcoholic fatty liver disease. Int. J. Mol. Sci..

[60] Lee DJ, Eor JY, Kwak M-J, Lee J, Kang AN, Mun D (2024). Metabolic regulation of longevity and immune response in *Caenorhabditis elegans* by ingestion of *Lacticaseibacillus rhamnosus* IDCC 3201 using multi-omics analysis. J. Mcrobiol. Botechnol..

[61] Kang A, Eor JY, Lee J, Kwak M-J, Lee DJ, Seo E (2025). *Lacticaseibacillus casei* IDCC 3451 alleviates cognitive and behavioral functions by reshaping the gut microbiome and regulating intestinal barrier integrity in chronic stress animal models. Curr. Res. Food Sci..

[62] Lee DJ, Eor JY, Kwak M-J, Lee J, Kang AN, Mun D (2025). Enhanced Longevity and Immunity in *Caenorhabditis elegans* through Ingestion of *Lactiplantibacillus plantarum* SKO-001: A Multi-Omics Study. Food Sci. Anim. Resour..

[63] Jang KB, Lee W, Park J, Kang A, Kim Y, Song M, *et al*. 2025. A commensal *Lactobacillus*-based probiotic consortium isolated from healthy pig feces alleviates weaning stress in nursery pigs via modulation of gut microbiota and metabolites. *J. Anim. Sci. Technol.* https://doi.org/10.5187/jast.2500384. 10.5187/jast.2500384

[64] Kinara E, Moturi J, Hosseindoust A, Mun JY, Tajudeen H, Ha SH (2025). Dietary supplementation of *Lactobacillus salivarius* in suckling and weanling piglets modulates intestinal microbiota, morphology and improves growth performance. J. Anim. Sci. Technol..

[65] Wei Y, Liang J, Su Y, Wang J, Amakye WK, Pan J (2021). The associations of the gut microbiome composition and short-chain fatty acid concentrations with body fat distribution in children. Clin. Nutr..

[66] Li T-T, Tong A-J, Liu Y-Y, Huang Z-R, Wan X-Z, Pan Y-Y (2019). Polyunsaturated fatty acids from microalgae Spirulina platensis modulates lipid metabolism disorders and gut microbiota in high-fat diet rats. Food Chem. Toxicol..

[67] Liu Q, Cai B-y, Zhu L-x, Xin X, Wang X, An Z-m, *et al*. 2020. Liraglutide modulates gut microbiome and attenuates nonalcoholic fatty liver in db/db mice. *Life Sci.* **261:**118457. https://doi.org/10.1016/j.lfs.2020.118457. 10.1016/j.lfs.2020.118457 32961235

[68] Yu L, Wang L, Yi H, Wu X (2020). Beneficial effects of LRP6-CRISPR on prevention of alcohol-related liver injury surpassed fecal microbiota transplant in a rat model. Gut Microbes.

[69] Miao J, Guo L, Cui H, Wang L, Zhu B, Lei J (2022). Er‐chen decoction alleviates high‐fat diet‐induced nonalcoholic fatty liver disease in rats through remodeling gut microbiota and regulating the serum metabolism. Evid. Based Complement. Alternat. Med..

[70] Feng J, Wu Y, Dai P, Wang D, Liu L, Chai B (2023). Gut microbial signatures of patients with primary hepatocellular carcinoma and their healthy first-degree relatives. J. Appl. Microbiol..

[71] Komiyama S, Yamada T, Takemura N, Kokudo N, Hase K, Kawamura YI (2021). Profiling of tumour-associated microbiota in human hepatocellular carcinoma. Sci. Rep..

[72] Zhai Q, Feng S, Arjan N, Chen W (2019). A next generation probiotic, *Akkermansia muciniphila*. Crit. Rev. Food Sci. Nutr..

[73] Kang A, Lee J, Eor JY, Kwak M-J, Kim Y-A, Oh S (2025). A comprehensive assessment of immunomodulatory potentials of Korean antler velvet extract in mouse and neurodegenerative *Caenorhabditis elegans* models. J. Anim. Sci. Technol..

[74] Segers A, de Vos WM (2023). Mode of action of *Akkermansia muciniphila* in the intestinal dialogue: role of extracellular proteins, metabolites and cell envelope components. Microbiome Res. Rep..

[75] Zhuge A, Li S, Lou P, Wu W, Wang K, Yuan Y (2022). Longitudinal 16S rRNA sequencing reveals relationships among alterations of gut microbiota and nonalcoholic fatty liver disease progression in mice. Microbiol. Spectr..

[76] Juárez-Fernández M, Porras D, Petrov P, Román-Sagüillo S, García-Mediavilla MV, Soluyanova P (2021). The synbiotic combination of *Akkermansia muciniphila* and quercetin ameliorates early obesity and NAFLD through gut microbiota reshaping and bile acid metabolism modulation. Antioxidants.

[77] Nian F, Wu L, Xia Q, Tian P, Ding C, Lu X (2023). *Akkermansia muciniphila* and Bifidobacterium bifidum prevent NAFLD by regulating FXR expression and gut microbiota. J. Clin. Transl. Hepatol..

[78] Qu D, Chen M, Zhu H, Liu X, Cui Y, Zhou W (2023). *Akkermansia muciniphila* and its outer membrane protein Amuc_1100 prevent high-fat diet-induced nonalcoholic fatty liver disease in mice. Biochem. Biophys. Res. Commun..

[79] Yang L, Hua M, Li D, Li F, He Y, Miao X (2025). Protective effects of ginseng soluble dietary fiber and its fecal microbiota extract on antibiotic-induced gut dysbiosis obese mice. J. Microbiol. Biotechnol..

[80] Dong X, Lin L, Liu L, Chen L, Shang N, Wang R (2025). Oral Administration of *Clostridium butyricum* Alleviates High-Fat Diet-Induced Obesity in Mice by Modulating Gut *Akkermansia muciniphila* Abundance via Direct Growth Promotion. J. Microbiol. Biotechnol..

[81] Kang L, Ma X, Yu F, Xu L, Lang L (2024). Dihydromyricetin alleviates non-alcoholic fatty liver disease by modulating gut microbiota and inflammatory signaling pathways. J. Microbiol. Biotechnol..

[82] Juanola O, Francés R, Caparrós E (2024). Exploring the relationship between liver disease, bacterial translocation, and dysbiosis: unveiling the gut-liver axis. Visc. Med..

