# Supplementary material for: Protective Effects of Fusarium venenatum-Based Mycoprotein against Metabolic Dysfunction-Associated Steatohepatitis via the Gut-Liver Axis
Source: J Microbiol Biotechnol. 2026 Apr 21;36:e2602028. doi: 10.4014/jmb.2602.02028 (PMC13102626; doi:10.4014/jmb.2602.02028)
Supplement: Supplementary file 1 [file jmb-36-e2602028-supple.pdf]

J. Microbiol. Biotechnol. 2026. 36: e2602028
https://doi.org/10.4014/jmb.2602.02028

Supplementary Figures

Protective effects of *Fusarium venenatum*-based mycoprotein against metabolic dysfunction-associated steatohepatitis via gut-liver axis modulation

Daniel Junpyo Lee[1], Daye Mun[2], Min-Geun Kang[1], Anna Kang[1], Youbin Choi[1], Eunsol Seo[1], Seon-hui Son[1], Jihyun Yoon[1], Arthur Junghun Kim[1], Min-Jin Kwak[3], Woo Kyun Kim[4], Minho Song[5], Sangnam Oh[6], and Younghoon Kim[1]*

[1]Department of Agricultural Biotechnology and Research Institute of Agriculture and Life Science, Seoul National University, Seoul 08826, Korea

[2]Division of Animal Bioscience and Integrated Biotechnology, College of Agriculture and Life Sciences, Gyeongsang National University, Jinju 52828, Korea

[3]Department of Forest Products and Biotechnology, Kookmin University, Seoul 02707, Korea

[4]Department of Poultry Science, University of Georgia, Athens, GA 30602, United States

[5]Department of Animal Science and Biotechnology, Chungnam National University, Daejeon 34134, Korea

[6]Department of Food and Nutrition, Jeonju University, Jeonju 55069, Korea

*To whom correspondence should be addressed: ykeys2584@snu.ac.kr

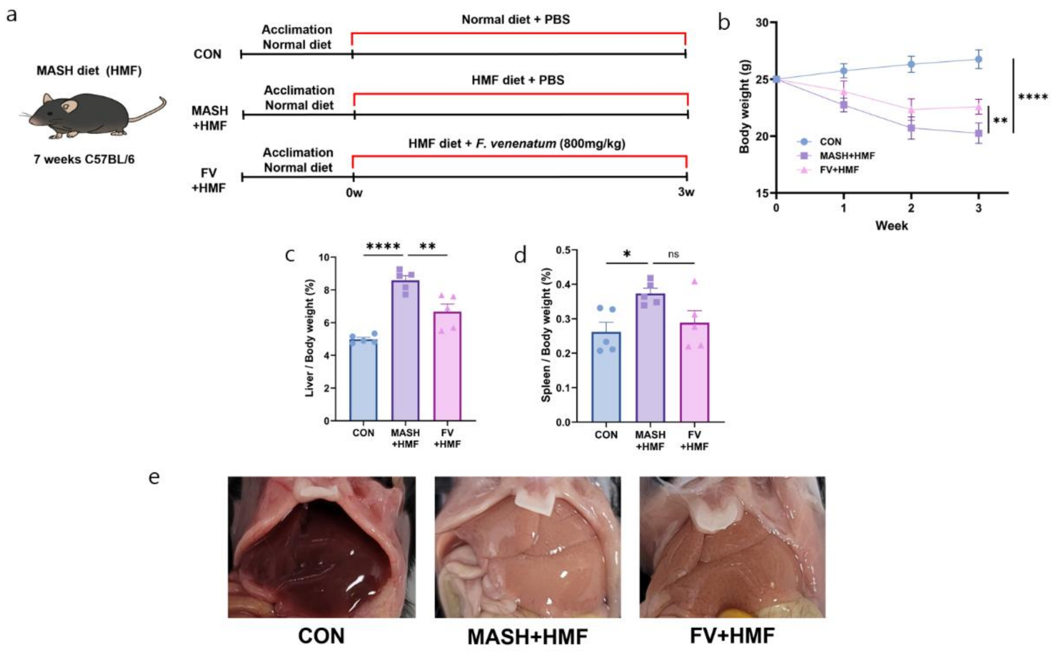

Fig. S1. ***F. venenatum*** **attenuated MASH progression in HMF diet-fed mice.**

(a) Experimental design. (b) Body weight change. (c) Liver weight. (d) Spleen weight. (e) Representative liver image. Data are expressed as means ± SEM. Statistical analysis was performed using one-way ANOVA. Differences were considered significant when p-value was below 0.05 (*), 0.01 (**), 0.001 (***), 0.0001 (****). CON group, received an oral administration of 200 μL of PBS along with a normal diet; MASH-HMF group, received an oral administration of 200 μL of PBS along with a HMF diet for MASH development; FV-HMF group, received an oral administration of *F. venenatum* at a dose of 800 mg/kg, suspended in 200 μL of PBS, along with a HMF diet for MASH development.

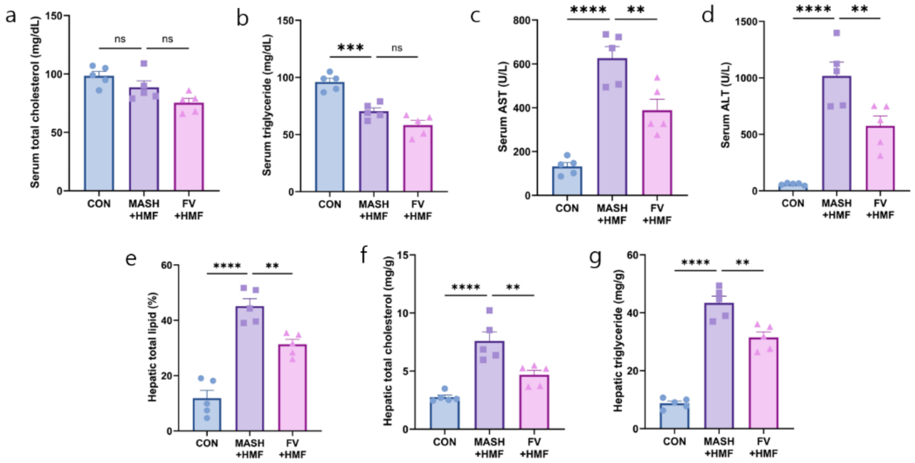

Fig. S2. ***F. venenatum*** **reduced liver toxicity and lipid accumulation in HMF diet-fed mice.**

(a) Serum total cholesterol. (b) Serum triglyceride. (c) Serum AST. (d) Serum ALT. (e) Hepatic total lipid. (f) Hepatic total cholesterol. (g) Hepatic triglyceride. Data are expressed as means ± SEM. Statistical analysis was performed using one-way ANOVA and differences were considered significant when p-value was below 0.05 (*), 0.01 (**), 0.001 (***), 0.0001 (****). CON group, received an oral administration of 200 μL of PBS along with a normal diet; MASH-HMF group, received an oral administration of 200 μL of PBS along with a HMF diet for MASH development; FV-HMF group, received an oral administration of *F. venenatum* at a dose of 800 mg/kg, suspended in 200 μL of PBS, along with a HMF diet for MASH development.

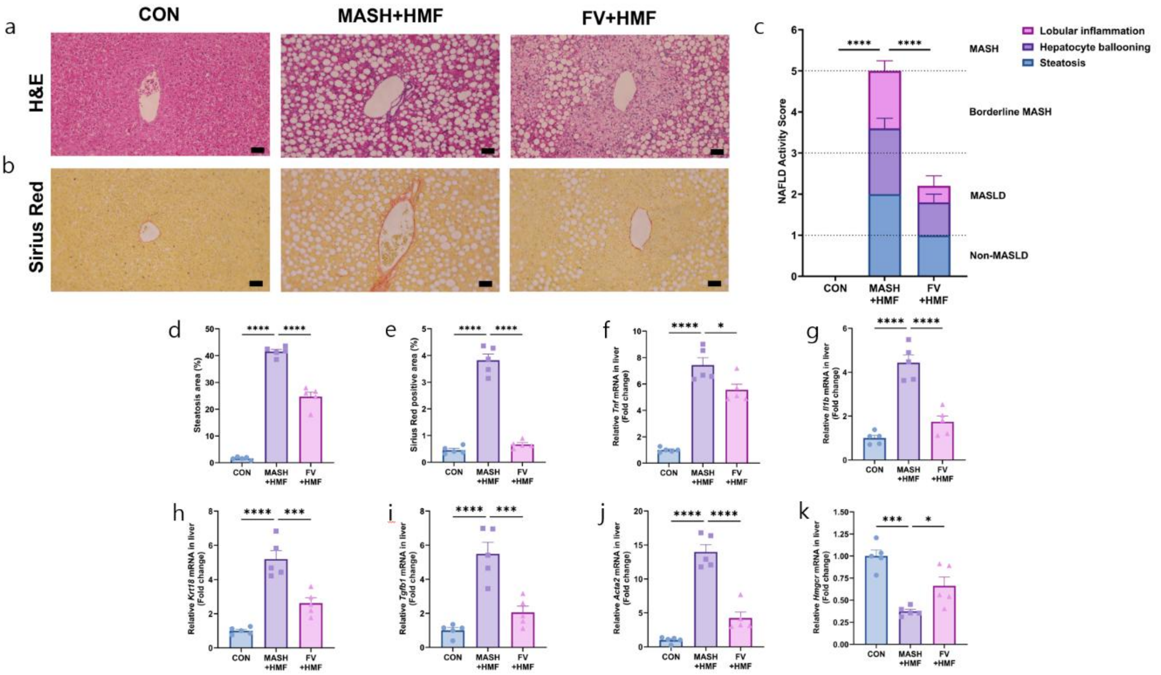

Fig. S3. *F. venenatum* ameliorated hepatic steatosis and fibrosis in HMF diet-fed mice.

(a) Representative images of hematoxylin and eosin-stained liver sections. Scale bar, 50 µm. (b) Representative images of Sirius Red-stained liver sections. Scale bar, 50 µm. (c) Nonalcoholic fatty liver disease activity score (NAS). (d) Hepatic steatosis area. (e) Hepatic fibrotic area. (f-k) Hepatic mRNA expression levels of genes associated with MASH progression. Inflammatory cytokines: (f) Tnf and (g) Il1b. Fibrosis-related genes: (h) *Krt18*, (i) *Tgfb1*, and (j) *Acta2*. Cholesterol metabolism-related genes: (k) *Hmgcr*. Gene expression was measured by qRT-PCR and normalized to the housekeeping gene *Hprt1*. Data are expressed as means ± SEM. Statistical analysis was performed using one-way ANOVA and differences were considered significant when p-value was below 0.05 (*), 0.01 (**), 0.001 (***), 0.0001 (****). CON group, received an oral administration of 200 µL of PBS along with a normal diet; MASH-HMF group, received an oral administration of 200 µL of PBS along with a HMF diet for MASH development; FV-HMF group, received an oral administration of *F. venenatum* at a dose of 800 mg/kg, suspended in 200 µL of PBS, along with a HMF diet for MASH development.

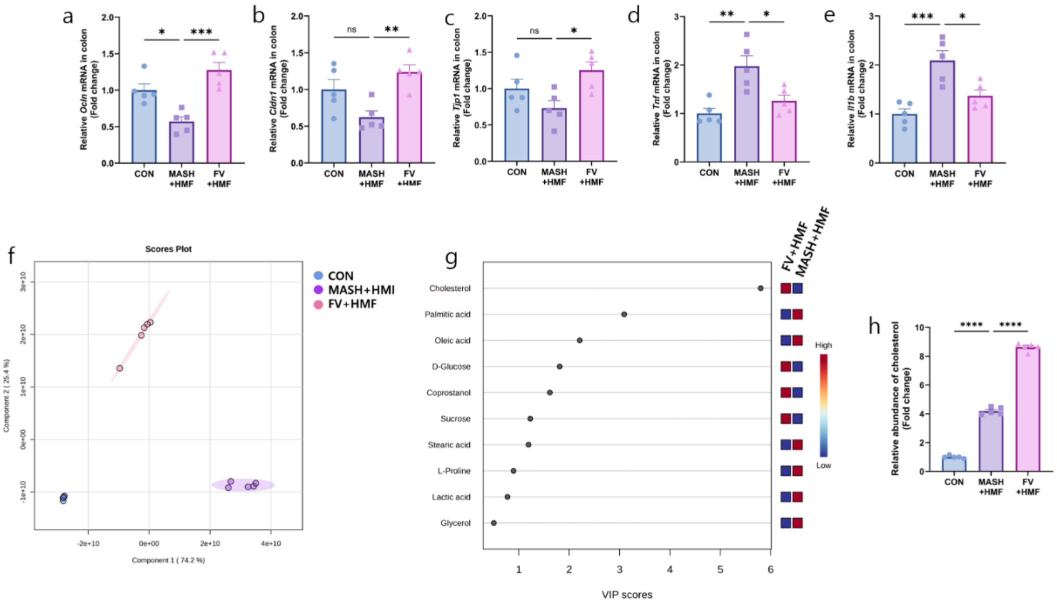

Fig. S4. ***F. venenatum*** **enhanced intestinal barrier integrity and cholesterol excretion in HMF diet-fed mice.**

(a-e) Intestinal mRNA expression levels of genes related to barrier integrity and inflammation. Barrier integrity-related genes: (a) *Ocln*, (b) *Cldn1*, and (c) *Tjp1*. Inflammatory cytokines: (d) *Tnf* and (e) *Il1b*. Gene expression was measured by qRT-PCR and normalized to the housekeeping gene *Hprt1*. (f) PLS-DA analysis of metabolites. (g) VIP score of PLS-DA analysis. (h) Relative abundance of cholesterol in feces. Data are expressed as means ± SEM. Statistical analysis was performed using one-way ANOVA and differences were considered significant when p-value was below 0.05 (*), 0.01 (**), 0.001 (***), 0.0001 (****). CON group, received an oral administration of 200 µL of PBS along with a normal diet; MASH-HMF group, received an oral administration of 200 µL of PBS along with a HMF diet for MASH development; FV-HMF group, received an oral administration of *F. venenatum* at a dose of 800 mg/kg, suspended in 200 µL of PBS, along with a HMF diet for MASH development. PLS-DA, partial least squares discriminant analysis; VIP, variable importance in projection.

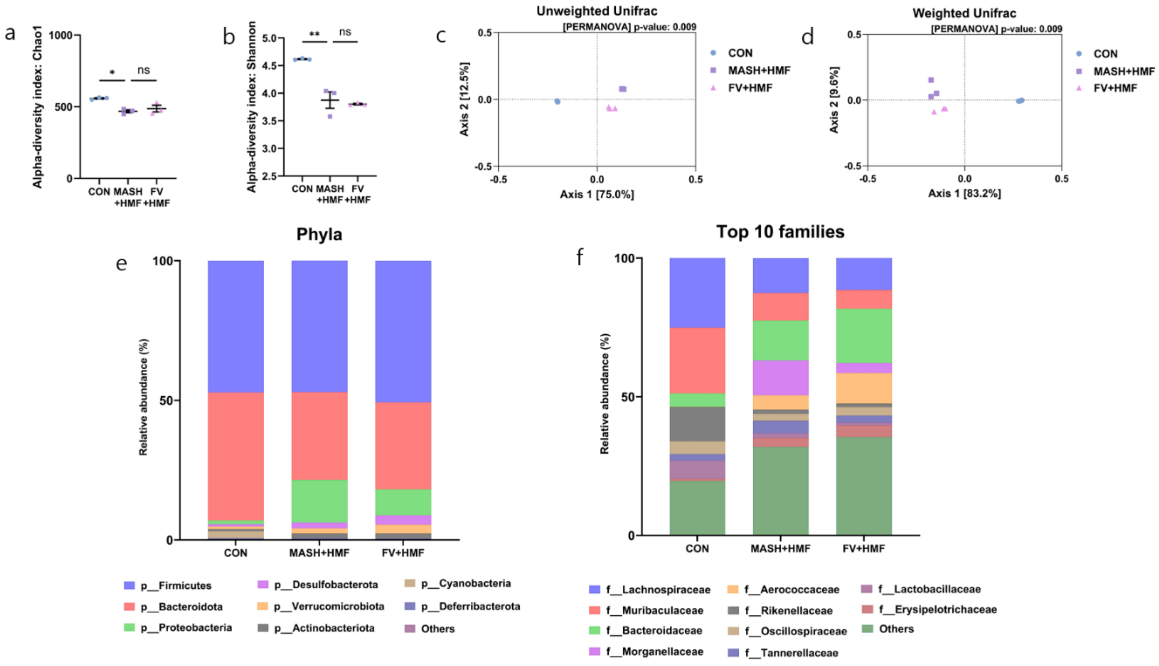

Fig. S5. *F. venenatum* modulated gut microbiota in HMF diet-fed mice.

(a) Comparison of alpha diversity based on Chao1 index values. (b) Comparison of alpha diversity based on Shannon index values. (c) Beta diversity analysis using unweighted UniFrac distances. (d) Beta diversity analysis using weighted UniFrac distances. (e) Relative abundance of microbial taxa at the phylum level. (f) Relative abundance of microbial taxa at the family level using the top 10 taxa. Statistical analysis was performed using one-way ANOVA and differences were considered significant when p-value was below 0.05 (*), 0.01 (**), 0.001 (***), 0.0001 (****). CON group, received an oral administration of 200 µL of PBS along with a normal diet; MASH-HMF group, received an oral administration of 200 µL of PBS along with a HMF diet for MASH development; FV-HMF group, received an oral administration of *F. venenatum* at a dose of 800 mg/kg, suspended in 200 µL of PBS, along with a HMF diet for MASH development.

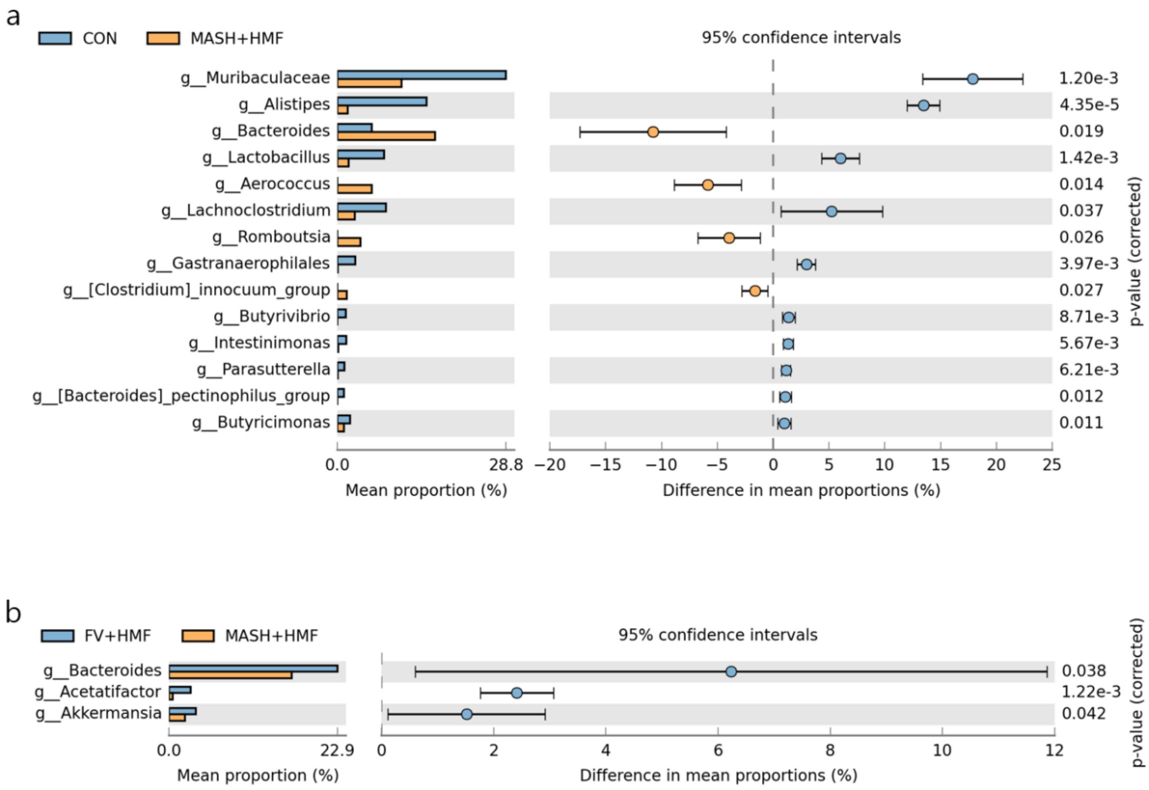

Fig. S6. *F. venenatum* altered specific gut microbial genera in HMF diet-fed mice.

(a) Differentially abundant genera between CON and MASH groups, identified using STAMP with Welch's t-test ($p < 0.05$). (b) Differentially abundant genera between FV and MASH groups, identified using STAMP with Welch's t-test ($p < 0.05$). Statistical analysis was performed using Welch's t-test. CON group, received an oral administration of 200 μL of PBS along with a normal diet; MASH-HMF group, received an oral administration of 200 μL of PBS along with a HMF diet for MASH development; FV-HMF group, received an oral administration of *F. venenatum* at a dose of 800 mg/kg, suspended in 200 μL of PBS, along with a HMF diet for MASH development.
